# Dysregulated gene expression in oocysts of *Plasmodium berghei* LAP mutants

**DOI:** 10.1016/j.molbiopara.2019.02.001

**Published:** 2019-04

**Authors:** Sadia Saeed, Ching-In Lau, Annie Z. Tremp, Tessa Crompton, Johannes T. Dessens

**Affiliations:** a Pathogen Molecular Biology Department, Faculty of Infectious and Tropical Diseases, London School of Hygiene & Tropical Medicine, Keppel Street, London, UK; b UCL Great Ormond Street Institute of Child Health, 30 Guilford Street, London, UK

**Keywords:** Plasmodium berghei, LCCL, Oocyst, Sporogony, Malaria transmission

## Abstract

Malaria parasite oocysts generate sporozoites by a process termed sporogony. Essential for successful sporogony of *Plasmodium berghei* in *Anopheles stephensi* mosquitoes is a complex of six LCCL lectin domain adhesive-like proteins (LAPs). LAP null mutant oocysts undergo growth and mitosis but fail to form sporozoites. At a cytological level, LAP null mutant oocyst development is indistinguishable from its wildtype counterparts for the first week, supporting the hypothesis that LAP null mutant oocysts develop normally before cytokinesis. We show here that LAP1 null mutant oocysts display highly reduced expression of sporozoite proteins and their transcription factors. Our findings indicate that events leading up to the cytokinesis defect in LAP null mutants occur early in oocyst development.

## Highlights

• Plasmodium berghei LAP null mutant oocysts display highly reduced levels of CSP.
• Transcription of other sporozoite genes and transcription factors is dysregulated.
• A minority oocyst population can bypass the developmental block in cytokinesis.

## Graphical abstract

**Figure:**
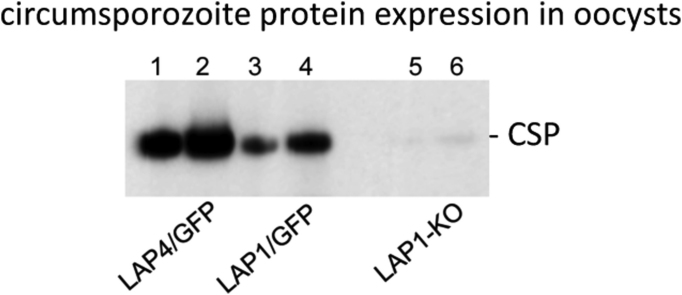

A large part of the *Plasmodium* life cycle takes place in the vector mosquito to achieve transmission of malaria parasites from one vertebrate host to the next. Development in the insect begins with the uptake of sexual stage precursor cells (gametocytes) from the vertebrate with the blood meal of a feeding mosquito, initiating a rapid process of gamete formation and fertilization inside the mosquito midgut. The resultant zygotes undergo meiosis and transform into motile elongated forms termed ookinetes, which traverse the midgut epithelium and then round up to form the oocysts. In the following weeks, young oocysts grow and divide by a process known as sporogony to generate hundreds of daughter cells named sporozoites. After egress from the oocyst, motile sporozoites invade and inhabit the salivary glands, and are transmissible to new hosts by mosquito bite to infect liver cells and initiate new malaria blood stage infections and complete the life cycle.

Critically involved in sporogony are a group of six modular proteins referred to as LCCL lectin domain adhesive-like proteins (LAPs) in *P. berghei* [1,2]. The LAPs possess multiple adhesive-like domains implicated in protein, lipid and carbohydrate binding, including the so-called ‘LCCL’ domain, a conserved protein module named after its founding proteins *Limulus* clotting factor C; cochlear protein Coch-5b2; and lung gestation protein Lgl1 [3]. The LAPs operate as a protein complex [4,5], and targeted disruption of any of the *lap* genes in *P. berghei* gives rise to a similar loss-of-function phenotypes typified by a failure of the oocyst to undergo cytokinesis and produce sporozoites, which is accompanied by increased oocyst growth [[6], [7], [8], [9], [10], [11]]. The *lap* genes are first and perhaps exclusively expressed in female gametocytes and are not expressed in sporozoites [9,10,12,13] indicating that their role is restricted to facilitating sporogony.

Another feature that the LAPs have in common is their subcellular localization in the crystalloid, a parasite organelle found uniquely in the ookinete and young oocyst life stages of the parasite [9,[12], [13], [14]]. The crystalloid organelle forms after fertilization, during zygote transformation into ookinete and then oocyst, by a process of active transport and assembly of endoplasmic reticulum (ER)-derived vesicles [8]. Besides the physical association of the LAPs and crystalloids, there is good evidence for a functional link between LAP expression, crystalloid biogenesis and sporogony. First, disruption of LAP1 or LAP3 in *P. berghei* abolishes formation of crystalloids [4,8,9] and this is probably also the case for the other LAP null mutants although this remains to be experimentally proven. Second, removal of the LCCL domain from LAP3 slows down the initial formation of the organelle, although normal crystalloids form by the time of oocyst transition with no discernible effect on sporogony [8]. Third, GFP tagging of LAP4 results in the formation of abnormal crystalloids, which is accompanied by reduced oocyst growth and earlier sporulation [11]. Thus, the LAPs' roles in sporogony could be indirect through facilitating the formation of the crystalloid organelle.

While LAP null mutant phenotypes in *P. berghei* are well characterised on a cellular level [[6], [7], [8],^11^,^15^], we know virtually nothing about the underlying molecular events. DNA staining and light microscopy show that development of LAP3 null mutant oocysts is indistinguishable from that of its wildtype counterparts for the first week after ookinete-to-oocyst transition [11], indicating that the initial phase of growth and mitosis progresses normally in LAP null mutants oocysts. Furthermore, a small percentage of LAP knockout oocysts in mosquitoes complete sporogony and generate morphologically normal sporozoites with circumsporozoite protein (CSP) expression and surface localisation [[7], [8], [9]], indicating that the LAP null mutants display normal expression of key sporozoite proteins. These combined observations led to the hypothesis that oocysts of LAP null mutants develop normally before cytokinesis, but then fail to pass a molecular checkpoint for progressing to sporogenesis. This study set out to test this hypothesis. We started by assessing CSP expression in whole oocyst populations of LAP1 (PBANKA_1035200) null mutant parasites. CSP is critically involved in cytokinesis as knockout of CSP expression gives rise to oocysts that fail to produce sporozoites [16], while knockdown of CSP expression leads to morphologically abnormal sporozoites [17]. *Anopheles stephensi* mosquitoes were infected as described [18] and maintained at 20 °C to allow mosquito stage parasite development. At 11 days post-infection oocyst-infected midguts were dissected and pooled. All guts in the samples were confirmed by light microscopy to have substantial numbers of oocysts (>20). We then carried out western blot analysis using anti-CSP antibodies. The results show that while *lap1*-positive parasite samples give rise to strong CSP signals, the CSP levels in equivalent *lap1*-negative samples are highly reduced (Fig. 1).

**Fig. 1:**
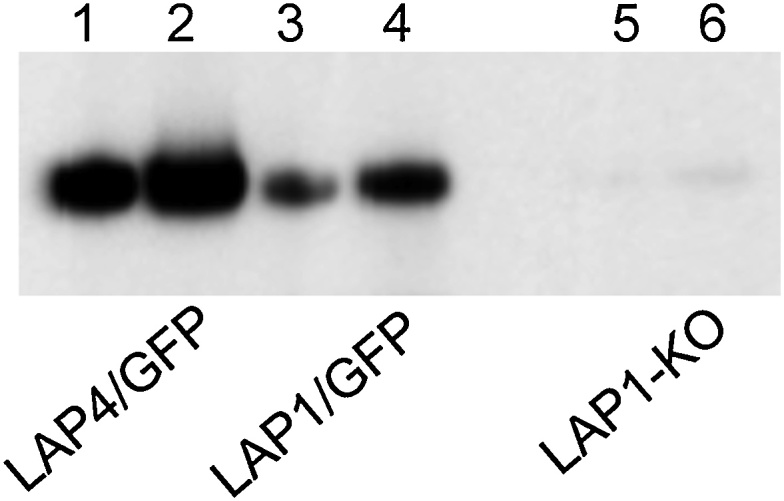
CSP expression in oocyst populations of LAP mutants assessed by western blot. Ten oocyst-infected midguts at 11 days post-infection were dissected and pooled. Samples were heated in reducing SDS-PAGE sample buffer at 70 °C for 10 min and proteins fractionated by electrophoresis through NuPage 4–12% Bis-Tris precast gels (Invitrogen) followed by transfer to PVDF membrane (Invitrogen). The gel was loaded with the equivalent of 2 (lanes 1, 3 and 5) or 4 (lanes 2, 4 and 6) oocyst-infected guts. The blot was developed using monoclonal antibody against CSP (3D11) as primary antibody, and goat anti mouse IgG conjugated to horseradish peroxidase (Invitrogen 81-6520) as secondary antibody, followed by chemiluminescence signal detection (ECL western blotting substrate, Pierce). Laboratory animal work was carried out in accordance with the United Kingdom Animals (Scientific Procedures) Act 1986 implementing European Directive 2010/63 for the protection of animals used for experimental purposes and was approved by the London School of Hygiene & Tropical Medicine ethical review committee and United Kingdom Home Office. Experiments were conducted in 6–8 weeks old female CD1 mice, specific pathogen free and maintained in filter cages. Animal welfare was assessed daily and animals were humanely killed upon reaching experimental or clinical endpoints. Mice were infected with parasites by intraperitoneal injection. Intra-erythrocytic parasitemia was monitored regularly by collecting of a small volume of blood from a superficial tail vein. Drugs were administered by intraperitoneal injection or where possible were supplied in drinking water. Parasitized blood was harvested by cardiac bleed under general anaesthesia without recovery.

In *P. berghei,* sporulation in oocyst populations is first detected at 8 days post-infection in a very low percentage of oocysts, and increases to reach peak levels a week later [11]. To investigate sporozoite gene expression in greater depth, we developed a reverse transcription quantitative real time PCR (RT-qPCR) method. Oocyst-infected midguts were dissected at day 7 and day 9 post-infection, representing time points before and during sporulation, respectively. Importantly, by day 7 post-infection the parasite-infected blood meal has been fully digested by the mosquito and the only parasite life cycle stages remaining in the insects are oocysts. For each time point, total RNA was extracted from approximately 30 dissected and pooled midguts using the RNeasy mini kit (Qiagen). To remove residual genomic DNA contamination, RNA samples were then subjected to DNase I treatment (amplification grade, Sigma) for 15 min at room temperature followed by heat inactivation of the enzyme. After a second RNeasy clean-up step to remove the DNase I, RNA samples were reverse transcribed with Moloney murine leukemia virus reverse transcriptase (RNase H minus, point mutation, Promega) in the presence of oligo(dT)_25_ at 50 °C for 1 h, followed by heat inactivation. The resultant cDNA samples were column purified using the QIAquick gel extraction kit (Qiagen) and eluted in water. Real time PCR of cDNA was carried out as described [19] using SYBR Green Supermix (Bio-Rad) and 0.5 μM of each primer. Where possible PCR primers were used that bridge intron splice junctions to ensure that amplification was cDNA-specific (Table 1). RT-qPCR reactions were carried out in three replicates and transcript levels were determined using the ΔCt-method normalised against housekeeping genes encoding cytoplasmic heat shock protein 90 (Hsp90) and cytoplasmic Hsp70 (Table 1). Ct values ranged between 20.7 and 23.2 for *hsp70* and between 22.2 and 23.8 for *hsp90* across samples and time points, supporting their abundant and constant expression and suitability as reference genes. Melt curve analysis was carried out after all PCR reactions to confirm that they produced single, specific products (data not shown). Besides LAP1-KO parasites that lack crystalloids and fail to sporulate, we included parasite line LAP4/GFP expressing GFP-tagged LAP4 (PBANKA_1319500) in the analysis, which forms abnormal crystalloids resulting in earlier sporozogenesis [11]. Parasite line LAP1/GFP, which displays normal crystalloid formation and sporogony [9], was used as the reference parasite.

**Table 1:** Properties of oligonucleotide primers used in RT-qPCR analysis.

Gene	ID	Sense primer	Antisense primer	Amplicon length (bp)	cDNA-specific
*hsp90*	PBANKA_0805700	CAACAGAGCAAAGATTACTGAATTAC	GGAGAGTTGGATACTGCATTG	156	yes
*hsp70*	PBANKA_0711900	AAAATTACAACCAAATGAAGTAGAAAC	ATTTTGGACATAATTGGAGAGC	140	no
*ap2-sp*	PBANKA_1329800	ATGATAATACAAGCAATAATAACAATAGTG	ACTGTGGAGATATATTACTCATTCCA	178	no
*ap2-sp2*	PBANKA_1001800	CCTAACACTACACGATATAAACATGAATAG	TCAAGCAATGCATGTTTACC	189	yes
*csp*	PBANKA_0403200	TGACGATTCTTATATCCCAAGC	CAGTATCAATATCTTCTAAGGTCAAATC	180	no
*imc1a*	PBANKA_0402600	CCTTATTATAGAACTGAGTTGAGCC	CAAAAATATTTACATTTCCATCTTCTC	180	yes
*trap*	PBANKA_1349800	TGGATGCATAGGTGTTGG	TTAGTTCCAGTCATTATCTTCAGG	150	no
*spect*	PBANKA_1355600	CATTGAAAACAACAAAACCC	AAATGATGTGACTTTTATTAAGTTCG	148	yes

RT-qPCR analysis revealed that transcripts of *csp* were readily detectable in both LAP1/GFP and LAP4/GFP oocysts by day 7, and by day 9 post-infection had markedly increased levels (Fig. 2) consistent with the increase of CSP expression during sporulation [20]. By contrast, in LAP1-KO oocysts *csp* mRNA was virtually undetectable at both time points (Fig. 2), consistent with the dramatic reduction of CSP protein expression in LAP1-KO oocysts (Fig. 1). The approximately 200-fold higher *csp* levels on day 7 post-infection in oocyst populations of the two sporulating parasite lines indicates that CSP expression comes on before cytokinesis, consistent with its demonstrated essential role in this process [17]. Like CSP, the inner membrane complex protein 1a (IMC1a), a main component of the sporozoite’s cortical membrane skeleton, is required during the process of cytokinesis: disruption of IMC1a results in only abnormally shaped sporozoites [21,22]. The pattern of *imc1a* transcription was similar to that of *csp* with mRNA levels showing significant downregulation at both time points in the LAP1 null mutant (Fig. 2). The expression patterns of *imc1a* and *csp* are consistent with the ability of LAP4/GFP oocysts and their wildtype (LAP1/GFP) counterparts to sporulate and form normal-shaped sporozoites.

**Fig. 2:**
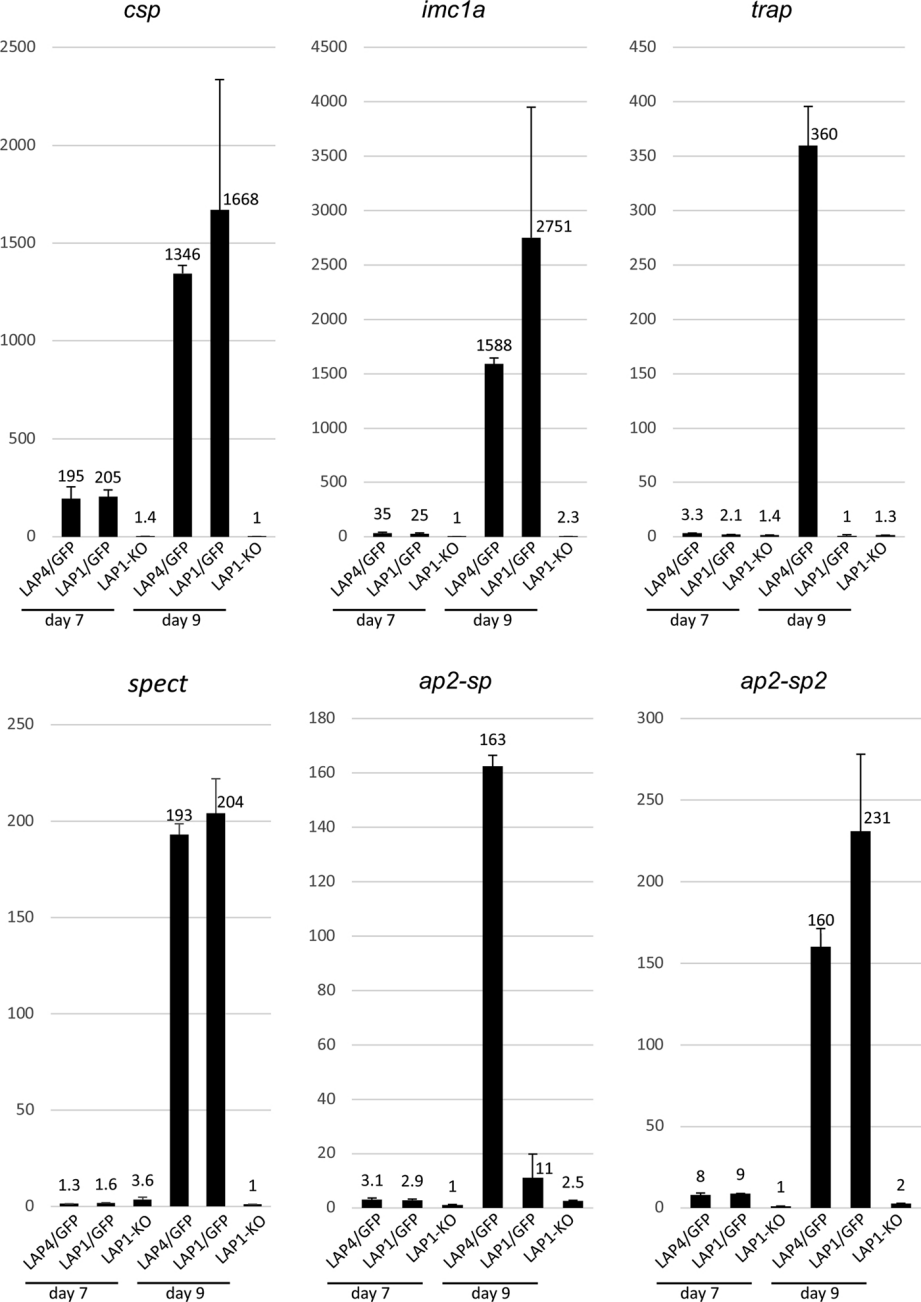
Sporozoite gene expression in LAP mutants assessed by RT-qPCR. Bar chart showing fold changes in messenger RNA levels of *csp, imc1a, trap, spect, a2-sp* and *ap2-sp2* genes relative to cytoplasmic *hsp90* and *hsp70* in oocyst populations of parasite lines LAP4/GFP, LAP1/GFP (wildtype reference) and LAP1-KO at 7 and 9 days post-infection of *Anopheles stephensi* mosquitoes. Lowest values were set to 1. Error bars denote standard deviations from three replicates. See Table 1 for gene IDs and primer properties.

We next assessed transcription of two sporozoite genes that are not required for cytokinesis in the oocyst. Thrombospondin-related adhesive protein (TRAP) has important functions after sporozoite formation including gliding motility *in vitro*, and sporozoite invasion of salivary glands and liver cells [23,24]. Transcript levels of *trap* in day 7 oocysts were low and comparable between the three parasite lines studied (Fig. 2), but by day 9 post-infection its mRNA levels had increased substantially in the LAP4/GFP mutant relative to both the LAP1/GFP and LAP1-KO parasites (Fig. 2). TRAP expression is reported to increase markedly after sporozoite formation [20] and, accordingly, the elevated levels of *trap* mRNA in day 9 oocysts is consistent with the higher level of sporulation in the LAP4/GFP oocyst population at this time point [11]. Sporozoite microneme protein essential for cell traversal (SPECT) is predominantly involved in sporozoite infectivity to the liver [25]. Transcript levels of *spect* were comparable between the three parasite lines in day 7 oocysts (Fig. 2). LAP1 null mutants showed no increase in *spect* transcription by 9 days post-infection, while the two sporulating parasite lines had markedly increased *spect* levels (approximately 200-fold) at the same time point (Fig. 2). This was surprising as SPECT protein is reported to be predominantly present in salivary gland sporozoites [25]. Our transcript analysis indicates that *spect* transcription in the oocyst population is not directly correlated with sporulation level. Collectively, the data indicate that there is a second (and stronger) peak in SPECT expression in the parasite after salivary gland colonisation.

We also assessed expression of the apetala2 (Ap2)-like transcription factors Ap2-SP and Ap2-SP2, which regulate expression of many sporozoite genes [26,27]. Knockout of Ap2-SP or Ap2-SP2 expression results in oocysts that undergo growth and mitosis, but do not produce sporozoites [26,27], a phenotype with similarities to the LAP null mutant oocyst phenotype. In day 7 oocysts, transcript levels of *ap2-sp* were reduced approximately 3-fold in LAP1-KO oocysts relative to the two sporulating parasite lines (Fig. 2). By day 9, transcript levels of *ap2-sp* had not significantly risen in the LAP1 null mutant, but had increased markedly in oocysts of the LAP4/GFP mutant compared to those of its wildtype counterpart (Fig. 2). This mRNA expression pattern is similar to that of *trap,* suggesting that Ap2-SP expression peaks in sporozoites after cytokinesis. This is in full agreement with Ap2-SP protein expression, which was reported to strongly increase in sporulating oocysts with the protein localizing to the nuclei of sporozoites [26]. The pattern of expression of *ap2-sp2* was distinct from that of *ap2-sp* resembling that of *csp* and *imc1a,* its expression again being highly reduced in the LAP1-KO parasite compared to its sporulating counterparts (Fig. 2).

Our findings show that relative to the housekeeping genes *hsp70* and *hsp90*, sporozoite gene expression in oocysts changes dramatically during their transition from the growth and mitosis phase to the cytokinesis phase. The data also provide the first molecular evidence that LAP1 null mutants display highly reduced expression levels of major sporozoite genes and transcription factors, which is already apparent before cytokinesis and could thus be causal with respect to the null mutant phenotype in the oocyst. These observations are poorly compatible with the hypothesis that oocysts of LAP null mutants develop normally before cytokinesis. The fact that some LAP null mutant oocysts generate morphologically normal sporozoites with CSP surface expression [7,9] thus implies that LAP null mutant parasite-infected mosquitoes can produce a minority oocyst population that overcome the developmental block in cytokinesis. Nonetheless, LAP null mutant sporozoites have not been successfully transmitted [[7], [8], [9]] indicating that they are non-infective as is indeed the case for sporozoites of the LAP4/GFP sporogony mutant [11]. Cytokinesis and sporozoite protein expression are closely linked and are controlled by genes and transcription factors that are distinct from those that control oocyst mitosis and growth [26,27]. The dysregulation of *ap2-sp* and *ap2-sp2* expression is likely be at least part responsible for the LAP mutant phenotypes with respect to oocyst differentiation and sporozoite development, given the considerable number and potentially overlapping repertoires of their target genes, and the complexity of their interactions with each other and within the larger network of transcriptional regulators [27]. Indeed, recent studies in the closely related parasite *P. yoelii* show that there are at least three other Ap2-type transcription factors expressed in oocysts during sporogony: Ap2-O2, Ap2-O3 and Ap2-O4 [28]. A key question is what controls the expression of the oocyst-specific transcription factors. This could involve other transcription factors that are expressed upstream of sporogony in young oocysts, ookinetes or even gametocytes. The RT-qPCR method described here will provide a useful tool for the further dissection of these convoluted developmental processes at the transcriptional level.
